# Trajectories of sustainable working life in nine Swedish residential regions: A longitudinal twin cohort study

**DOI:** 10.1002/1348-9585.12406

**Published:** 2023-05-22

**Authors:** Auriba Raza, Mo Wang, Jurgita Narusyte, Pia Svedberg, Annina Ropponen

**Affiliations:** ^1^ Division of Insurance Medicine, Department of Clinical Neuroscience Karolinska Institutet Stockholm Sweden; ^2^ Center for Epidemiology and Community Medicine Stockholm County Council Stockholm Sweden; ^3^ Finnish Institute of Occupational Health Työterveyslaitos Finland

**Keywords:** group‐based trajectory model, regions, sickness absence, sustainable working life, twins

## Abstract

**Objective:**

To investigate trajectories of sustainable working life (SWL, ie, no interruptions or transitions in working life due to sickness absence (SA), disability pension (DP), or unemployment) in Swedish residential regions using a population‐based twin cohort, while assessing sociodemographics and twin pair similarity.

**Methods:**

Sample of 60 998 twins born in 1925–1958. SWL was assessed through main labor market status in each year in 1998–2016 based on > 180 days with SA/DP, > 180 days with unemployment, or >half of yearly income from old‐age pension for not in SWL, and employment (in paid work and did not fulfill the criteria SA/DP, unemployment, or old‐age pension) for SWL. Residential regions were classified into nine groups based on Swedish municipalities. Group‐based trajectory models and multinomial logistic regression were applied separately for all regions.

**Results:**

In all regions, the largest trajectory group was sustainable working life. Three to four trajectory groups developed toward unsustainable working life with different exit points from sustainable working life. A small proportion were grouped with partial stable or increase in sustainable working life. Increased age, being a woman, <12 years of education, and history of unstable working life increased, and being married and twin pair similarity decreased the likelihood of belonging to trajectories toward unsustainable working life.

**Conclusions:**

In all regions, most of the individuals followed a sustainable working life trajectory. A reasonable proportion of individuals followed trajectories developing toward unsustainable working life. The influence of sociodemographic and familial factors on trajectory groups was similar in all regions.

## 1 INTRODUCTION

Sustainable working life refers to the process of living and working conditions that support people to continue working for an extended working life.^1^ It is an important concept considering an increasing life expectancy and has gained attention worldwide.^1^ Sustainable working life aligns with the definition of employability which is an individual's (perceived) ability to obtain and maintain employment throughout his/her career.^2^ However, register studies utilizing national data of participation in working life and social benefits provide an approach for sustainable working life as that is detailed and without reporting bias.^3^, ^4^, ^5^ Influential factor for sustainable working life,^6^ apart from working conditions^6^, ^7^ has been health.^6^, ^8^ Nevertheless, residential regions, where working and living conditions differ depending on for example local demographics, occupational composition, local attitudes, differences in education, disparities in transport facilities, socioeconomic strata, lack of work, and disparities in health care system,^9^, ^10^, ^11^, ^12^ are crucial for sustainable working life but studied rarely so far.

Working life is usually characterized by changes in employment, living, and working conditions and potentially consequent changes between employment statuses.^13^ Such changes might be independent or be a part of a cluster, since each change in working life may affect the likelihood of other changes, for example, unemployment or sickness absence might be linked with low future employability.^13^, ^14^ These changes can be directly or indirectly influenced by regional characteristics and consequently have an impact on sustainable working life. Previously, studies have reported higher risk of sickness absence and disability pension for individuals living in rural or semi‐rural municipalities compared to urban municipalities or towns.^9^, ^10^, ^12^, ^15^, ^16^ However, to the best of our knowledge, studies are lacking for the differences and similarities between residential regions on sustainable working life. Hence, a study with evaluation of longitudinal trajectories of sustainable working life over time while comparing different residential regions would add to the knowledge needed for policymakers, social security, and occupational health care to target actions in regions with highest needs.

Sociodemographic characteristics of the residential region can play a role for sustainable working life. Based on results of unsustainable working life (ie, interruptions or transitions in working life due to sickness absence or disability pension), being a woman has shown to be associated with an increased risk of sickness absence or disability pension.^9^, ^16^, ^17^ Also, older age has been linked with the associations between residential regions and the risk of disability pension or sickness absence.^9^, ^16^, ^17^ Moreover, regions with a higher share of manual jobs, higher level of self‐reported mechanical, physical, and chemical exposure have been associated with high levels of sickness absence.^15^ Furthermore, genetics may also play a role in the associations of regions and sustainable working life since genetics explains 26%–50% of the individual differences in sickness absence and disability pension.^18^, ^19^ Therefore, a study investigating sustainable working life over the life course while also assessing sociodemographic factors and twin pair similarity (zygosity) could contribute to understanding the association between residential regions and sustainable working life.

The aim was to investigate trajectories of sustainable working life in Swedish residential regions using a population‐based twin cohort with a follow‐up from 1999 to 2016. Based on the hypothesis that residential regions related to larger cities might have better opportunities for sustainable working life than the smaller ones, the aim was to investigate the regions separately. We further aimed to assess the role of various sociodemographic factors and twin pair similarity for belonging to the regional trajectory groups.

## 2 METHODS

The data were derived from the Swedish Twin study of Disability pension and Sickness absence (STODS) and include 119 907 twins born in Sweden between 1925 and 1958 identified through the Swedish Twin Registry (STR).^20^ We restricted the study sample to those who at the baseline year 31 December 1998 were alive, living in Sweden, had data on regions, were 18 to 67 years old, and employed.^4^ There were 60 998 twin individuals in the final sample with complete data at the baseline including 14 860 monozygotic (MZ) and 18 118 same‐sexed dizygotic (DZ) twins.

### 2.1 Sustainable working life

The data on time of emigration (censoring), annual days of unemployment, employment, and old age pension for 1994–2016 were from Statistics Sweden database Longitudinal Integration Database for Health Insurance and Labour Market Studies (LISA).^21^ Annual net days with sickness absence and disability pension were from the Swedish Social Insurance Agency MiDAS‐database. Data for date of death was obtained from Cause of Death Register maintained by National Board of Health and Welfare for censoring. Individuals who died or emigrated during follow‐up period were included in the analysis until the year of death or emigration. Censoring applied also when turning 68 years of age. During the follow‐up from 1999 to 2016, sustainable working life was assessed through main labor market status in each year of follow‐up using information on sickness absence (SA) or disability pension (DP) (>180 days with SA/DP benefits), unemployment (>180 days with unemployment benefits), old‐age pension (more than half of yearly income from old‐age pension), and employment (ie, in paid work and did not fulfill the criteria SA/DP, unemployment, or old‐age pension). For statistical analyses, we coded sustainable working life variable as 1 if employed and 0 otherwise.

### 2.2 Regions

The type of living area, that is, regions, was from LISA.^21^ We applied the classification of Swedish municipalities by the Swedish Association of Local Authorities and Regions to data.^22^ The municipalities were categorized into nine groups based on structural parameters such as population and commuting patterns:

1. Large cities—municipalities with a population of at least 200 000 inhabitants with at least 200 000 inhabitants in the largest urban area.
2. Commuting municipalities near large cities—municipalities where more than 40% of the working population commute to work in a large city or municipality near a large city.
3. Medium‐sized towns—municipalities with a population of at least 50 000 inhabitants with at least 40 000 inhabitants in the largest urban area.
4. Commuting municipalities near medium‐sized towns—municipalities where more than 40% of the working population commute to work in a medium‐sized town.
5. Commuting municipalities with a low commuting rate near medium‐sized towns—municipalities where <40% of the working population commute to work in a medium‐sized town.
6. Small towns—municipalities with a population of at least 15 000 inhabitants in the largest urban area.
7. Commuting municipalities near small towns—municipalities where more than 30% of the working population commute to work in a small town/urban area or more than 30% of the employed day population lives in another municipality.
8. Rural municipalities – municipalities with a population of <15 000 inhabitants in the largest urban area, very low commuting rate (<30%).
9. Rural municipalities with a visitor industry—municipalities in rural area that fulfill at least two criteria for visitor industry, that is, number of overnight stays, retail, restaurant, or hotel turnover per head of population.

### 2.3 Factors of interest

Data on age, sex, education level, marital status, occupation sector at baseline in 1998 were from LISA.^21^ Age was used as a continuous variable in the statistical analyses. Sex, men (reference) and women. Years of education were categorized as 0–9, 10–12, and >12 years (reference). Occupation sector was categorized as public (reference), private, and other, including those not classified yet. History of sustainable working life before baseline in 1998 (ie, 1994–1997) was categorized as stable employment (ie, being employed all years 1994–1997; reference); changed and status in 1997 employed; stable unemployment; changed and status in 1997 unemployed; stable sickness absence or disability pension; changed and status in 1997 sickness absence or disability pension. For familial factors, we used zygosity, that is, MZ, and DZ same‐sexed twins (reference).

### 2.4 Statistical methods

Descriptive statistics (mean with standard deviation [SD]) and frequencies with percentage (%) were calculated for the final sample and across residential regions. Group‐based trajectory model (GBTM), an application of finite mixture modeling, was used to estimate change over time in a repeatedly measured outcome. GBTM is designed to identify groups with similar longitudinal response patterns over time^23^, ^24^ and enables to differentiate and describe subpopulations that exist within a studied population.^25^ The GBTM assists identification of the timing of transition from one phase to another, namely sustainable working life phase to unsustainable working life phase.^24^ The GBTM assumes that in each group, individuals have the same sustainable working life pattern. In this study, GBTM with the ‘traj’ in Stata was used to distinguish different trajectories for sustainable working life from 1999 to 2016 using annual status of sustainable working life, that is, employment coded as 1 or unemployment, sickness absence/disability pension, old age pension coded as 0. A prior decision for the smallest trajectory group was set at ≥5%. Lowest values in Bayesian information criterion (BIC), Akaike Information Criteria (AIC), and highest in average posterior probability (APP) were used to confirm the goodness of fit.^26^ Measures of goodness of fit for group‐based trajectory models are presented in Table S1.

Based on the best fitted model for each region, multinomial logistic regression was applied to elucidate the association of sociodemographic factors and zygosity with the identified trajectories. For the assessment of twin pair similarity, zygosity was added to the regression models to study monozygotic twins' likelihood of belonging to trajectory groups compared to dizygotic twins. Twin pair similarity assumed that twins share genetic factors (100% for MZ and ≈50% for DZ) and family background that includes home and social family environment primarily during childhood.

All analyses were performed using Stata version 14.

## 3 RESULTS

Descriptive statistics of the study population are presented in Table S1. In regions 1, 2, 3, 8, and 9, women were in majority. About three‐fourths of the study population had <12 years of education both among the total sample and across the regions. More than half of the individuals worked in the private sector, and most (70% or more) had a stable history of working life.

**FIGURE 1:**
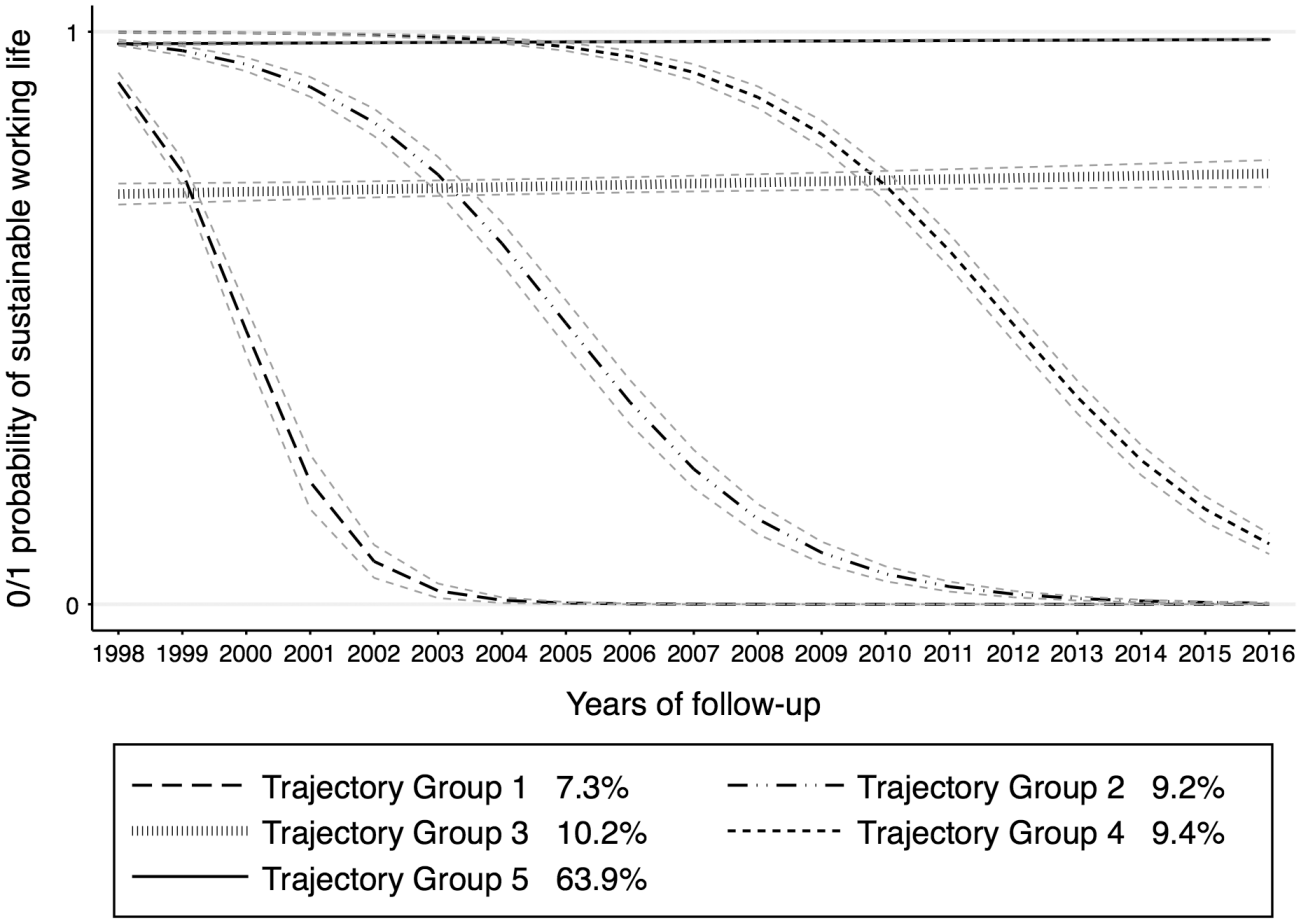
Trajectory groups of sustainable working life in region 1, Large cities—municipalities with a population of at least 200 000 inhabitants with at least 200 000 inhabitants in the largest urban area.

For the region 1 (ie, large cities), five sustainable working life trajectories were identified with 64% of the individuals in the stable sustainable working life in trajectory group (TG) 5 (Figure 1). Individuals (10%) belonging to TG3 had a partial stable sustainable working life. While TG1, the smallest group, had an early sharp decline in sustainable working life and then stayed in unsustainable working life throughout the follow‐up period. Sustainable working life of TG2 started to decline after 2 years of follow‐up, while TG4 showed a later decline in sustainable working life.

Increasing age seemed to have influence on the likelihood of belonging to all the trajectory groups with highest estimates observed for TG1 and 2 compared to the reference TG 5, that is, stable sustainable working life (Table 1). Being a woman was associated with increased likelihood of belonging to TGs 1 and 3. Married individuals were less likely to belong to TGs 1 and 2, while had an increased likelihood of belonging to TG4. Individuals with <12 years of education had an increased likelihood of belonging to all the trajectory groups. Individuals with an history of unstable working life were more likely to belong to TGs 1, 2, and 3 (Table 1).

**TABLE 1:** Relative risk ratios (RRR) with 95% confidence intervals (CI) for sociodemographic factors in relation to sustainable working life trajectory groups in nine residential regions of Sweden.

	Trajectory groups					
	1	2	3	4	5	6
	RRR (95%CI)	RRR (95%CI)	RRR (95%CI)	RRR (95%CI)	RRR (95%CI)	RRR (95%CI)
Region 1						
5‐cluster model					**1** (ref)	
Age	**1.19 (1.17–1.20)**	**1.13 (1.12–1.15)**	**1.02 (1.01–1.03)**	**1.08 (1.07–1.09)**		
Sex						
Men	1 (ref)	1 (ref)	1 (ref)	1 (ref)		
Women	**1.23 (1.01–1.49)**	0.98 (0.83–1.16)	**1.51 (1.28–1.77)**	1.07 (0.92–1.25)		
Civil status						
Neither married nor in relationship	1 (ref)	1 (ref)	1 (ref)	1 (ref)		
Married or in partnership	**0.68 (0.57–0.82)**	**0.82 (0.70–0.96)**	0.83 (0.68–1.01)	**1.19 (1.02–1.40)**		
Education						
0–9 years	**2.72 (2.08–3.56)**	**2.16 (1.69–2.75)**	**2.39 (1.86–3.06)**	**1.88 (1.48–2.38)**		
10–12 years	**2.09 (1.69–2.57)**	**1.65 (1.38–1.98)**	**1.70 (1.42–2.03)**	**1.43 (1.21–1.69)**		
>12 years	1 (ref)	1 (ref)	1 (ref)	1 (ref)		
Occupation sector						
Public	1 (ref)	1 (ref)	1 (ref)	1 (ref)		
Private	0.97 (0.80–1.19)	1.08 (0.90–1.29)	0.99 (0.84–1.17)	1.08 (0.92–1.27)		
Other	1.17 (0.72–1.89)	1.24 (0.82–1.90)	1.12 (0.77–1.64)	0.83 (0.54–1.30)		
History of SWL						
Stable employment	1 (ref)		1 (ref)	1 (ref)		
Changed, status in 1997 employed	**3.21 (2.42–4.25)**	**1.85 (1.45–2.37)**	**2.35 (1.90–2.91)**	1.00 (0.78–1.27)		
Stable unemployment	**4.72 (2.08–0.70)**	**3.62 (1.83–7.16)**	**6.84 (4.50–10.40)**	0.74 (0.29–1.89)		
Changed, status in 1997 unemployed	**5.80 (3.73–9.00)**	**2.80 (1.88–4.16)**	**4.23 (3.24–5.53)**	**1.46 (1.00–2.15)**		
Changed, status in 1997 SA/DP	**19.03 (6.92–52.33)**	**2.26 (0.61–8.40)**	2.81 (0.79–0.10)	0.63 (0.08–5.27)		
Zygosity						
Dizygotic	1 (ref)	1 (ref)	1 (ref)	1 (ref)		
Monozygotic	0.86 (0.69–1.08)	0.84 (0.68–1.03)	0.96 (0.78–1.19)	**0.79 (0.64–0.97)**		

Region 2						
6‐cluster model	1	2	3	4 (ref)	5	6
Age	**1.22 (1.20–1.25)**	**1.17 (1.16–1.19)**	**1.15 (1.14–1.16)**		**1.07 (1.07–1.08)**	**1.02 (1.01–1.03)**
Sex						
Men	1 (ref)	1 (ref)	1 (ref)		1 (ref)	1 (ref)
Women	**1.31 (1.07–1.61)**	**1.31 (1.11–1.55)**	1.10 (0.94–1.30)		**1.25 (1.06–1.47)**	**1.58 (1.34–1.87)**
Civil status						
Neither married nor in relationship	1 (ref)	1 (ref)	1 (ref)		1 (ref)	1 (ref)
Married or in partnership	0.86 (0.71–1.05)	**0.80 (0.67–0.94)**	0.92 (0.78–1.07)		**1.22 (1.03–1.45)**	0.84 (0.71–1.01)
Education						
0–9 years	**1.96 (1.48–2.60)**	**1.98 (1.55–2.52)**	**1.67 (1.33–2.11)**		1.20 (0.93–1.53)	**1.98 (1.55–2.53)**
10–12 years	**1.70 (1.34–2.14)**	**1.82 (1.50–2.21)**	**1.64 (1.37–1.96)**		**1.36 (1.14–1.63)**	**1.34 (1.11–1.62)**
>12 years	1 (ref)	1 (ref)	1 (ref)		1 (ref)	1 (ref)
Occupation sector						
Public	1 (ref)	1 (ref)	1 (ref)		1 (ref)	1 (ref)
Private	0.97 (0.78–1.20)	1.11 (0.93–1.33)	1.09 (.92–1.30)		1.08 (0.91–1.29)	1.13 (0.94–1.34)
Other	1.17 (0.71–1.91)	0.99 (0.61–1.61)	0.92 (0.58–1.47)		1.15 (0.73–1.81)	0.63 (0.36–1.09)
History of SWL						
Stable employment	1 (ref)	1 (ref)	1 (ref)		1 (ref)	1 (ref)
Changed, status in 1997 employed	**2.71 (1.99–3.70)**	**2.03 (1.56–2.65)**	**1.07 (0.81–1.42)**		0.97 (0.74–1.28)	**2.15 (1.73–2.68)**
Stable unemployment	**6.70 (2.95–15.20)**	**5.35 (2.53–11.33)**	**3.61 (1.79–7.26)**		1.80 (0.73–4.46)	**7.25 (4.52–1.64)**
Changed, status in 1997 unemployed	**7.57 (4.25–13.49)**	**5.54 (3.44–8.92)**	1.55 (0.87–2.78)		0.76 (0.41–1.41)	**4.88 (3.65–6.52)**
Changed, status in 1997 SA/DP	**5.80 (1.99–6.94)**	**4.37 (1.28–4.94)**	1.14 (0.24–5.50)		0.72 (0.09–5.86)	**4.11 (1.29–13.08)**
Zygosity						
Dizygotic	1 (ref)	1 (ref)	1 (ref)		1 (ref)	1 (ref)
Monozygotic	1.13 (0.89–1.44)	1.06 (0.86–1.30)	0.89 (0.73–1.08)		0.88 (0.71–1.08)	1.05 (0.86–1.29)

Region 3					
5‐cluster model	1	2	3	4	5 (ref)
Age	**1.19 (1.18–1.20)**	**1.14 (1.13–1.15)**	**1.03 (1.02–1.04)**	**1.09 (1.08–1.09)**	
Sex					
Men	1 (ref)	1 (ref)	1 (ref)	1 (ref)	
Women	**1.20 (1.04–1.39)**	**1.18 (1.03–1.35)**	**1.69 (1.46–1.95)**	**1.16 (1.02–1.31)**	
Civil status					
Neither married nor in relationship	1 (ref)	1 (ref)	1 (ref)	1 (ref)	
Married or in partnership	**0.70 (0.62–0.80)**	0.88 (0.77–1.00)	**0.66 (0.56–0.77)**	**0.97 (0.85–1.10)**	
Education					
0–9 years	**2.23 (1.84–2.71)**	**1.97 (1.63–2.39)**	**1.78 (1.44–2.19)**	**1.68 (1.39–2.02)**	
10–12 years	**1.72 (1.45–2.02)**	**1.82 (1.56–2.13)**	**1.26 (1.07–1.47)**	**1.68 (1.45–1.93)**	
>12 years	1 (ref)	1 (ref)	1 (ref)	1 (ref)	
Occupation sector					
Public	1 (ref)	1 (ref)	1 (ref)	1 (ref)	
Private	0.89 (0.76–1.04)	1.02 (0.88–1.18)	**1.19 (1.02–1.38)**	1.10 (0.96–1.25)	
Other	1.09 (0.76–1.57)	1.16 (0.83–1.63)	1.07 (0.76–1.51)	0.75 (0.52–1.09)	
History of SWL					
Stable employment	1 (ref)	1 (ref)	1 (ref)	1 (ref)	
Changed, status in 1997 employed	**2.66 (2.14–3.32)**	**1.47 (1.19–1.81)**	**2.21 (1.84–2.65)**	1.01 (0.83–1.24)	
Stable unemployment	**8.44 (4.91–14.52)**	**3.59 (2.10–6.11)**	**8.38 (6.02–1.66)**	1.67 (0.97–2.87)	
Changed, status in 1997 unemployed	**5.03 (3.44–7.36)**	**1.94 (1.31–2.89)**	**3.57 (2.81–4.55)**	1.18 (0.83–1.67)	
Changed, status in 1997 SA/DP	**10.48 (3.65–30.16)**	1.81 (0.51–6.40)	**5.50 (2.04–4.87)**	0.44 (0.05–3.57)	
Zygosity					
Dizygotic	1 (ref)	1 (ref)	1 (ref)	1 (ref)	
Monozygotic	**0.70 (0.59–0.83)**	**0.74 (0.63–0.87)**	1.12 (0.95–1.33)	**0.72 (0.61–0.85)**	

Statistically significant RRR with 95% CI in boldface.

Abbreviations: DP, disability pension; SA, sickness absence; WL, sustainable working life.

**FIGURE 2:**
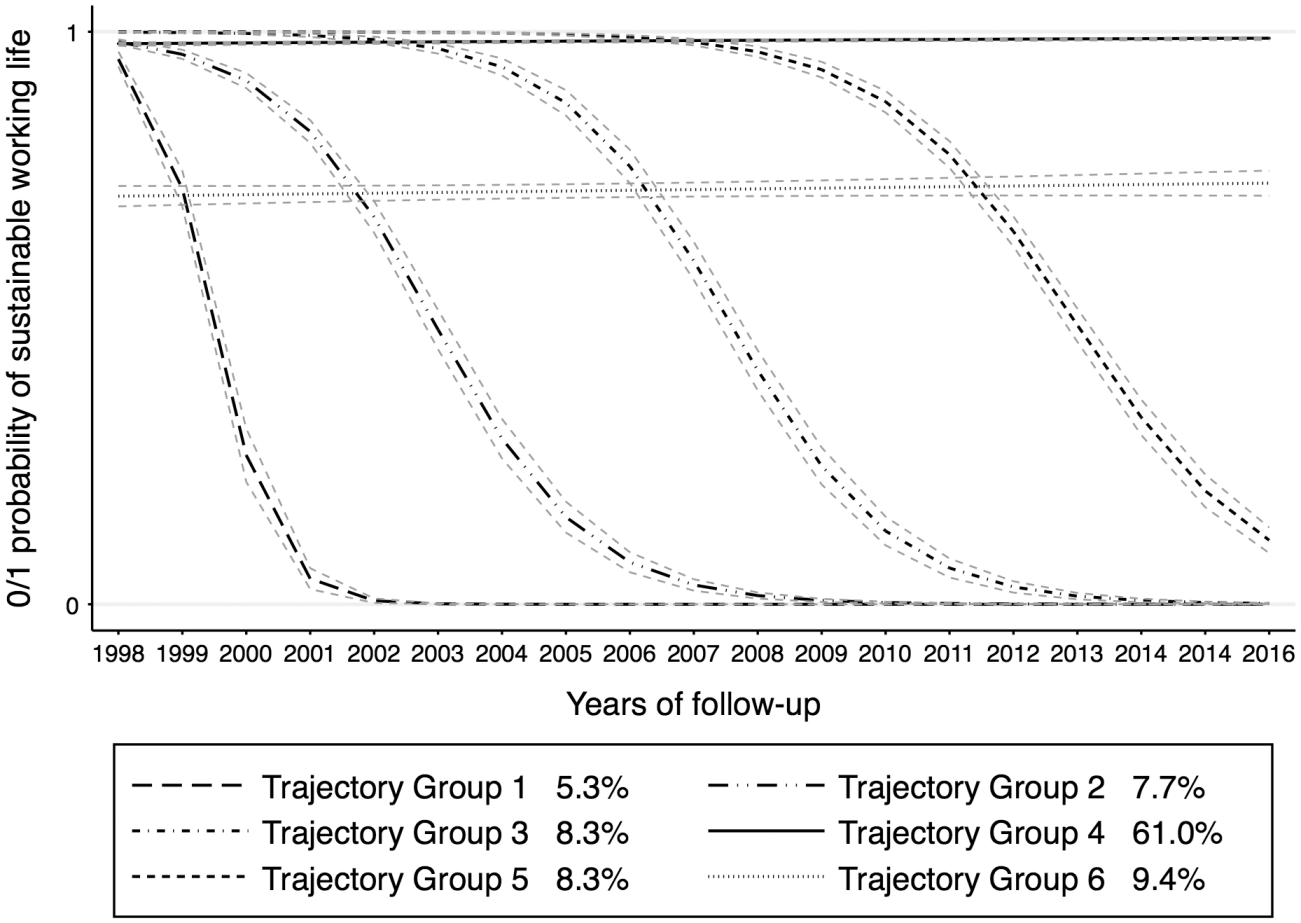
Trajectory groups of sustainable working life in region 2, Commuting municipalities near large cities—municipalities where more than 40% of the working population commute to work in a large city or municipality near a large city.

The region 2 (ie, commuting municipalities near large cities, Figure 2) had three TGs 2, 3, and 5 which showed similar development with different exit points from sustainable working life. Similar TGs were observed for region 8 (rural municipalities, Figure S5). In region 2, age increased the likelihood of belonging to TGs as the region 1. Being a woman increased the likelihood of belonging to all the TGs except for TG3. Married individuals were likely to belong to TG5; however, no such effect is observed for other TGs. Low education affected belonging to TGs that were developing toward unsustainable working life. History of unstable working life increased the likelihood of belonging to TGs (Table 1). While for the region 8, increased age, low education, and history of unstable working life had an influence on being a member of TGs (Table S2).

**FIGURE 3:**
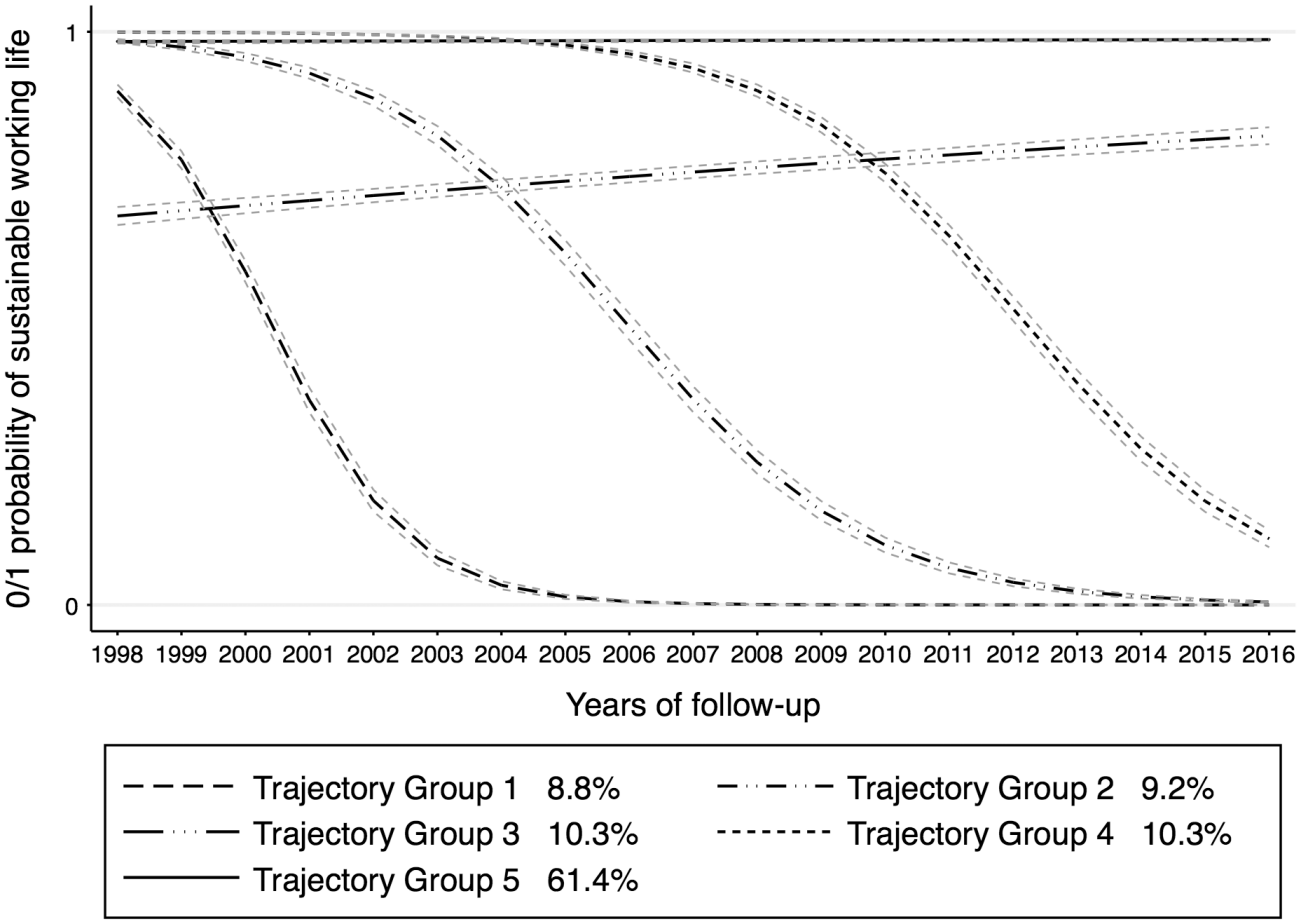
Trajectory groups of sustainable working life in region 3, Medium‐sized towns—municipalities with a population of at least 50 000 inhabitants with at least 40 000 inhabitants in the largest urban area.

For region 3 (medium‐sized towns), we observed similar TGs as region 1 except TG3 that showed stable increase in sustainable working life (Figure 3). Being a woman was observed to increase the likelihood of belonging to TGs besides age. Married individuals were less likely to belong to any of the TGs in region 3. Individuals working in a private sector were more likely to belong to TG3 that showed increase in sustainable working life (Table 1). Monozygotic twins were less likely to belong to all TGs except TG3.

Regions 4, 5, 6, and 9 (Figures S1–S3, and S6) had similar TGs and sociodemographic factors and being monozygotic twin had similar effect on belonging to TGs as region 3 (Table S2). However, in region 9 (rural municipalities with a visitor industry) being a woman, married, and having low education were not associated with the likelihood of belonging to TGs. Region 7 (commuting municipalities near small towns) had one additional TG4 that showed a stable decrease in sustainable working life (Figure S4), while other TGs showed similar development as regions 4, 5, 6, and 9. Increasing age and history of unstable working life was observed to have an influence on being a member of TGs in the region 9 (Table S2).

## 4 DISCUSSION

This study was based on a large Swedish population‐based cohort of twins to investigate regional trajectories of sustainable working life with a long follow‐up of 18 years and to assess the role of sociodemographic factors and twin pair similarity in belonging to the regional trajectory groups. To the best of our knowledge, this is among the first studies to investigate this. We identified five to six trajectory groups in all regions that had a similar trend: the largest trajectory group had a stable sustainable working life, another trajectory group had partial stable sustainable working life, third with early steep decline in sustainable working life with a stay in unsustainable working life state throughout the follow‐up, whereas two or three trajectory groups gradually developed toward unsustainable working life with different exit points from sustainable working life, and yet a last trajectory group had a stable increase in sustainable working life. For belonging to these trajectory groups, increasing age, being a woman, <12 years of education, and a history of unstable working life were associated with higher likelihood of belonging to trajectory groups developing toward unsustainable life in all regions. Instead, being married and twin pair similarity (being monozygotic twin) had a less likelihood of belonging to these trajectory groups in nearly all regions.

### 4.1 Regional sustainable working life trajectories

The Swedish welfare system is guided by principles of universalism and solidarity with a superseding aim to promote social equity^27^ and this was reflected in our results where we did not observe major differences in trajectories of sustainable working life between Swedish residential living regions over 18 years of follow‐up. However, we identified some trajectory groups that eventually developed toward unsustainable working life. These might suggest predictive actions in early life course especially for women, older individuals, those with low education or earlier periods of unsustainable working life (ie, sickness absence or unemployment). The identification of regional sustainable working life trajectories and recognizing the elucidating sociodemographic and familial factors are relevant in the Swedish context since the government has emphasized on sustainable working life in its work environment strategy for 2021–2025.^28^ Furthermore, the results of this study might also be relevant in effective targeting of policies, regulations, and practices on promoting sustainable working life.

### 4.2 Influential factors for trajectory group memberships

Demographic composition, educational level, occupational sector etc., were assumed to affect the likelihood of belonging to the trajectories of sustainable working life. Our results indicated that increased age, being a woman, having low education, and history of sickness absence and unemployment played a role for the likelihood of individuals belonging to trajectories developing toward unsustainable working life. Our results are supported by earlier findings of associations between being woman, older ages and sickness absence and disability pension.^9^, ^16^, ^17^ The mechanism linking education with sickness absence has been that higher education would be determinant for better health and work life functioning.^29^ Our results on level of education are in agreement with earlier studies that reported less years of education to be associated with higher prevalence of disability pension.^29^ Furthermore, history of sickness absence is a significant predictor of future sickness absence and studies have reported that earlier sickness absence increases the risk of transitioning from work to sickness absence, from sickness absence to unemployment, from work to unemployment, and from work to disability pension.^14^ This is in line with our findings of history of unstable working life being associated with increased likelihood of being a member of trajectories developing toward unsustainable working life. Nevertheless, we also observed that married individuals had less likelihood of belonging to nearly all trajectory groups compared to the reference group of stable sustainable working life. Previous studies have observed mixed effects of marital status alone and/or in combination with household composition on sickness absence and disability pension, suggesting a complex relationship.^17^, ^30^

### 4.3 Familial confounding in sustainable working life

Furthermore, twin pair similarity (zygosity) played a role in the likelihood of belonging to trajectory groups. Hence, an assumption remains that familial confounding (ie, genetics, and shared environment in the childhood) might play a role for sustainable working life. Twins are known to be representative of the general population^31^ and findings from earlier studies of partially based on the same data but considering sickness absences and disability pension have been consistent with studies on singleton population.^16^, ^32^

### 4.4 Strengths and weaknesses

Strengths of this study include the large sample size, long follow‐up, no dropout, and the availability of the high‐quality data from Swedish national registries. Having data from recent years is another strength since neither regional differences in sustainable working life nor sociodemographic characteristics of regions are stable over time.^33^ No studies are without limitations, and we need to address the fact that utilizing register data limited us from evaluating important work‐related factors, lifestyle factors, physician's certification practices, differences in attitudes and norms regarding the use of welfare system with relevance for regions and sustainable working life.^34^ Furthermore, we lacked data on sickness absence shorter than 14 days which might be relevant and should be tackled in further studies if possible. Yet a limitation might be the generalization of the results to other countries except the Nordic region where welfare and social security system are relatively similar in comparison to Sweden.

## 5 CONCLUSIONS

In all regions, most of the individuals followed a sustainable working life trajectory. A reasonable proportion of individuals followed trajectories developing toward unsustainable working life, while a small proportion were grouped in trajectories following either stable or increase in sustainable working life. The influence of sociodemographic and familial factors on trajectory groups was similar in all residential regions.

## DISCLOSURE

*Approval of the research protocol*: The study protocol was designed and performed according to the principles of the Helsinki Declaration. The ethical vetting was performed and approved by the Regional Ethical Review Board of Stockholm, Sweden (Dnr: 2007/524‐31, 2010/1346‐32/5, 2014/311‐32, 2015/1809‐32, 2017/128‐32). For this project, the Regional Ethical Review Board of Stockholm stated that the consent to participate was not applicable in these type of large register studies. None of the authors had access to any kind of identifying information. *Informed consent*: Not applicable. *Registry and the Registration No. of study*: Not applicable. *Animal studies*: Not applicable. *Conflict of interest*: None declared.

## Supporting information


Table S1–S2
Figure S1–S6

## Data Availability

The data that support the findings of this study are available from the original sources: the Swedish Twin Registry, Statistics Sweden, Swedish Social Insurance Agency and the Swedish National Board of Health and Welfare. Restrictions apply to the availability of the data used in this study based on the Swedish Twin project Of Disability pension and Sickness absence (STODS), which were used with ethical permission for the current study and therefore are not publicly available.
