# Supplementary material for: Trajectories of sustainable working life in nine Swedish residential regions: A longitudinal twin cohort study
Source: J Occup Health. 2023 May 22;65(1):e12406. doi: 10.1002/1348-9585.12406 (PMC10203348; doi:10.1002/1348-9585.12406)
Supplement: Supplementary file 1 — Table S1–S2 Figure S1–S6 [file JOH2-65-e12406-s001.docx]

|  | **Whole sample, n (%)** | **Regions, n (%)** | | | | | | | | |
| --- | --- | --- | --- | --- | --- | --- | --- | --- | --- | --- |
|  |  | **1** | **2** | **3** | **4** | **5** | **6** | **7** | **8** | **9** |
| **N** | 60998 (100) | 9410 (100) | 10520 (100) | 14469 (100) | 5200 (100) | 4258 (100) | 8648 (100) | 4162 (100) | 3392 (100) | 939 (100) |
| **Age** |  |  |  |  |  |  |  |  |  |  |
| 18-27 | 10993 (18) | 2036 (21) | 1779 (22) | 2984 (17) | 859 (17) | 685 (16) | 1359 (16) | 620 (15) | 521 (15) | 150 (16) |
| 28-37 | 14431 (24) | 2636 (28) | 2632 (25) | 3374 (23) | 1143 (22) | 907 (21) | 1993 (23) | 846 (20) | 710 (21) | 190 (20) |
| 37-47 | 14645 (24) | 2136 (23) | 2472 (24) | 3318 (23) | 1301 (25) | 1125 (26) | 2149 (25) | 1058 (25) | 869 (26) | 217 (23) |
| 48-57 | 15829 (26) | 1974 (21) | 2809 (27) | 3627 (25) | 1412 (27) | 1156 (27) | 2352 (27) | 1241 (30) | 962 (28) | 296 (32) |
| 58-65 | 5070 (8) | 624 (7) | 819 (8) | 1155 (8) | 483 (9) | 385 (9) | 793 (9) | 396 (10) | 329 (10) | 86 (9) |
| **Sex** |  |  |  |  |  |  |  |  |  |  |
| Women | 30454 (50) | 4936 (52) | 5,373 (51) | 7281 (50) | 2473 (48) | 2110 (49) | 4180 (48) | 1920 (46) | 1707 (50) | 474 (51) |
| **Marital status** |  |  |  |  |  |  |  |  |  |  |
| Married | 27704 (45) | 3050 (32) | 5190 (49) | 6338 (44) | 2691 (52) | 2051 (48) | 4230 (49) | 2164 (52) | 1567 (46) | 423 (45) |
| **Education** |  |  |  |  |  |  |  |  |  |  |
| 0-9 years | 12892 (21) | 1305 (14) | 1,914 (18) | 2645 (18) | 1430 (28) | 1120 (26) | 2020 (23) | 1274 (31) | 912 (27) | 272 (29) |
| 10-12 years | 30555 (50) | 4114 (44) | 5251 (50) | 6889 (48) | 2815 (54) | 2315 (54) | 4601 (53) | 2168 (52) | 1900 (56) | 502 (53) |
| >12 years | 17477 (29) | 3966 (42) | 3339 (32) | 4925 (34) | 948 (18) | 820 (19) | 2019 (23) | 717 (17) | 579 (17) | 164 (18) |
| **Occupational sector** |  |  |  |  |  |  |  |  |  |  |
| Public | 26152 (43) | 3840 (41) | 4114 (39) | 6714 (46) | 2088 (40) | 1882 (44) | 3885 (45) | 1587 (38) | 1640 (48) | 402 (43) |
| Private | 32787 (54) | 5186 (55) | 6088 (58) | 7214 (50) | 2956 (57) | 2267 (53) | 4463 (52) | 2456 (59) | 1664 (49) | 493 (53) |
| Other | 2059 (3) | 384 (4) | 318 (3) | 541 (4) | 156 (3) | 109 (3) | 300 (4) | 119 (3) | 88 (3) | 44 (5) |
| **History of sustainable working life** |  |  |  |  |  |  |  |  |  |  |
| Stable employment | 44791 (73) | 6649 (71) | 7966 (76) | 10511 (73) | 3823 (74) | 3145 (74) | 6461 (75) | 3141 (76) | 2443 (72) | 652 (70) |
| Changed, status in 1997 employed | 9218 (15) | 1657 (18) | 1433 (14) | 2285 (16) | 743 (14) | 621 (15) | 1251 (15) | 562 (14) | 498 (15) | 168 (18) |
| Stable unemployment | 1016 (2) | 158 (2) | 128 (1) | 249 (2) | 96 (2) | 63 (2) | 150 (2) | 69 (2) | 79 (2) | 24 (3) |
| Changed, status in 1997 unemployed | 3026 (5) | 520 (5) | 424 (4) | 733 (5) | 280 (5) | 214 (5) | 401 (5) | 200 (5) | 200 (6) | 54 (6) |
| Stable sickness absence or disability pension | 25 (0) | <10 | <10 | <10 | <10 | <10 | <10 | <10 | <10 | <10 |
| Changed, status in 1997 SA/DP | 215 (0.4) | 34 (0.4) | 28 (0.3) | 43 (0.3) | 28 (0.5) | 16 (0.4) | 17 (0.2) | 19 (1) | 25 (1) | <10 |
| **Zygosity** |  |  |  |  |  |  |  |  |  |  |
| Monozygotic | 14860 (24) | 2330 (25) | 2727 (26) | 3646 (25) | 1226 (24) | 995 (23) | 2035 (24) | 943 (23) | 732 (22) | 226 (24) |
| Dizygotic | 18118 (30) | 2687 (29) | 3063 (29) | 4276 (29) | 1602 (31) | 1294 (30) | 2596 (30) | 1262 (30) | 1060 (31) | 278 (30) |

**Supplemental Table S1** Descriptive characteristics of the study population at the baseline year 1998, stratified on residential regions

Supplemental Table S2 Measure of Goodness of fit of group-based trajectory models for sustainable working life in residential regions in Sweden

| **Model** | **Smallest group** | | **BIC** | **AIC** |  |
| --- | --- | --- | --- | --- | --- |
|  | **N** | **%** |  |  | **APP** |
| Region 1 |  |  |  |  |  |
| 2-cluster model | 2131 | 22.90 | -42411.00 | -42393.13 | 0.95 |
| 3-cluster model | 1142 | 11.87 | -39070.05 | -39041.46 | 0.92 |
| 4-cluster model | 1167 | 10.71 | -37868.28 | -37828.97 | 0.86 |
| **5-cluster model** | **701** | **7.30** | **-36843.74** | **-36793.71** | **0.91** |
| 6-cluster model | 356 | 4.76 | -36405.29 | -36344.54 | 0.88 |
| Region 2 |  |  |  |  |  |
| 2-cluster model | 2353 | 22.7 | -46466.39 | -46448.24 | 0.95 |
| 3-cluster model | 1349 | 12.48 | -42836.08 | -42807.04 | 0.92 |
| 4-cluster model | 752 | 9.32 | -41282.75 | -41242.82 | 0.89 |
| 5-cluster model | 998 | 9.04 | -39902.76 | -39851.95 | 0.90 |
| **6-cluster model** | **578** | **5.28** | **-39379.62** | **-39317.91** | **0.90** |
| Region 3 |  |  |  |  |  |
| 2-cluster model | 3179 | 22.20 | -65520.09 | -65501.15 | 0.95 |
| 3-cluster model | 1705 | 11.53 | -60391.19 | -60360.87 | 0.92 |
| 4-cluster model | 1349 | 10.76 | -58053.51 | -58011.82 | 0.85 |
| **5-cluster model** | **1385** | **8.57** | **-56572.77** | **-56519.71** | **0.82** |
| 6-cluster model | 366 | 2.66 | -55903.92 | -55839.50 | 0.87 |
| Region 4 |  |  |  |  |  |
| 2-cluster model | 1195 | 22.95 | -24304.95 | -24288.57 | 0.95 |
| 3-cluster model | 775 | 14.38 | -22364.05 | -22337.83 | 0.92 |
| 4-cluster model | 492 | 8.89 | -21680.84 | -21644.79 | 0.91 |
| **5-cluster model** | **363** | **8.66** | **-20775.62** | **-20729.74** | **0.91** |
| 6-cluster model | 196 | 4.44 | -20572.23 | -20516.51 | 0.87 |
| 7-cluster model | 146 | 3.46 | -20200.30 | -20134.75 | 0.88 |
| Region 5 |  |  |  |  |  |
| 2-cluster model | 966 | 22.94 | -19732.35 | -19716.46 | 0.96 |
| 3-cluster model | 636 | 14.29 | -18147.77 | -18122.35 | 0.93 |
| 4-cluster model | 365 | 10.06 | -17323.88 | -17288.92 | 0.87 |
| **5-cluster model** | **443** | **10.02** | **-16705.18** | **-16660.68** | **0.92** |
| 6-cluster model | 157 | 4.69 | -16644.45 | -16590.43 | 0.89 |
| Region 6 |  |  |  |  |  |
| 2-cluster model | 1965 | 23.02 | -40061.38 | -40043.72 | 0.95 |
| 3-cluster model | 1116 | 12.61 | -36793.96 | -36765.70 | 0.93 |
| 4-cluster model | 787 | 8.60 | -35755.43 | -35716.58 | 0.91 |
| **5-cluster model** | **780** | **8.41** | **-34229.94** | **-34180.49** | **0.90** |
| 6-cluster model | 252 | 3.63 | -33864.43 | -33804.38 | 0.86 |
| Region 7 |  |  |  |  |  |
| 2-cluster model | 968 | 23.50 | -18966.04 | -18950.21 | 0.96 |
| 3-cluster model | 610 | 14.02 | -17267.52 | -17242.19 | 0.92 |
| 4-cluster model | 310 | 9.42 | -16642.53 | -16607.70 | 0.87 |
| 5-cluster model | 305 | 9.19 | -16023.39 | -15979.06 | 0.91 |
| 6-cluster model | 330 | 7.32 | -15824.32 | -15770.49 | 0.82 |
| **7-cluster model** | **2404** | **5.16** | **-15715.57** | **-15652.24** | **0.95** |
| Region 8 |  |  |  |  |  |
| 2-cluster model | 834 | 24.87 | -17339.26 | -17323.94 | 0.96 |
| 3-cluster model | 494 | 14.21 | -16036.98 | -16012.46 | 0.93 |
| 4-cluster model | 240 | 7.55 | -15390.88 | -15357.17 | 0.89 |
| 5-cluster model | 276 | 9.21 | -14863.24 | -14820.33 | 0.90 |
| **6-cluster model** | **282** | **7.59** | **-14640.13** | **-14588.03** | **0.89** |
| 7-cluster model | 123 | 4.28 | -14433.32 | -14372.03 | 0.80 |
| Region 9 |  |  |  |  |  |
| 2-cluster model | 228 | 24.06 | -5007.73 | -4995.63 | 0.94 |
| 3-cluster model | 128 | 13.65 | -4574.80 | -4555.42 | 0.95 |
| 4-cluster model | 71 | 9.52 | -4438.77 | -4412.13 | 0.80 |
| **5-cluster model** | **97** | **9.64** | **-4303.37** | **-4269.47** | **0.80** |
| 6-cluster model | 34 | 4.64 | -4275.04 | -4233.87 | 0.89 |


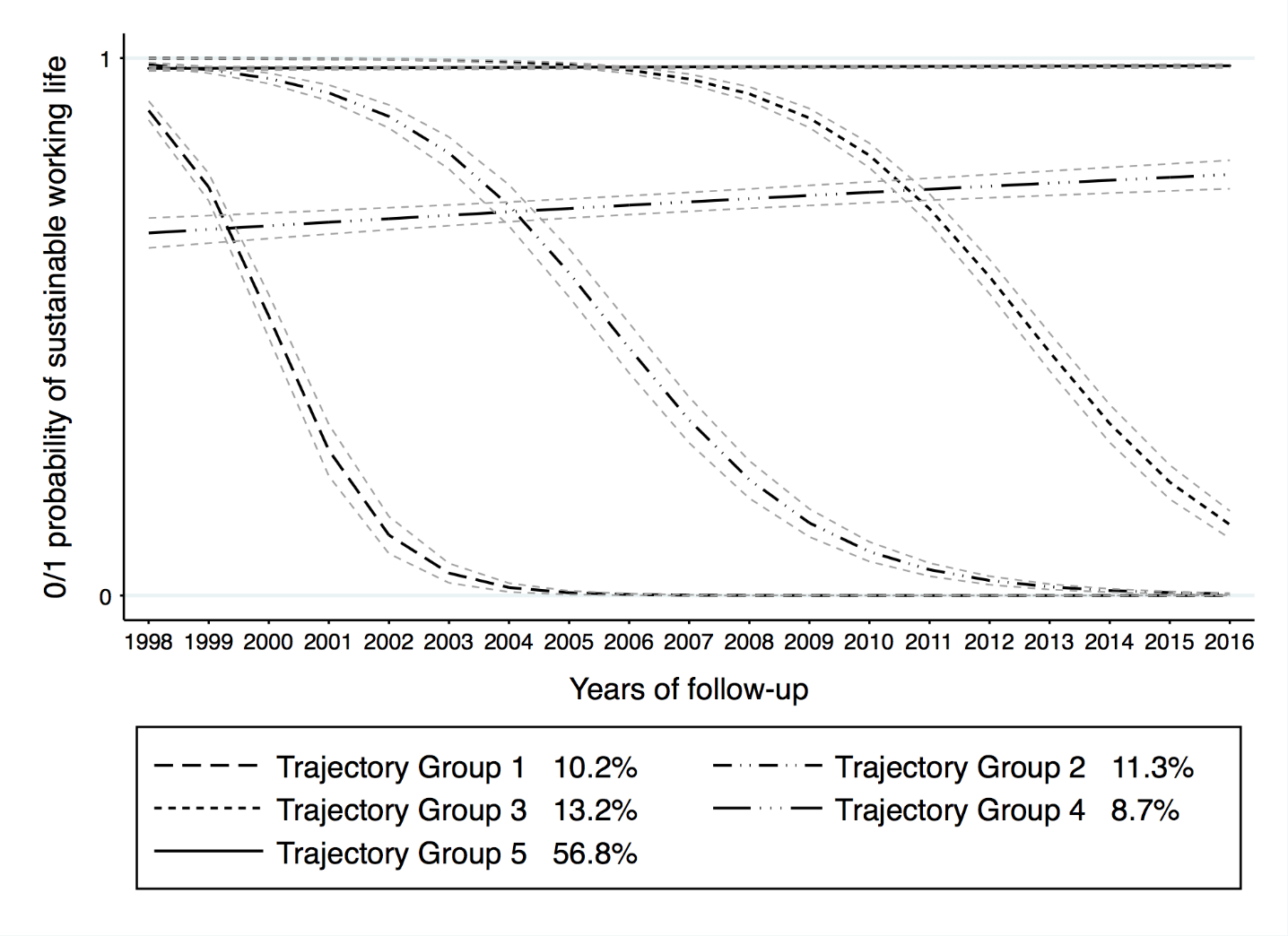


**Supplemental Figure S1** Trajectory groups of sustainable working life in region 4, Commuting municipalities near medium-sized towns - municipalities where more than 40 % of the working population commute to work in a medium-sized town.


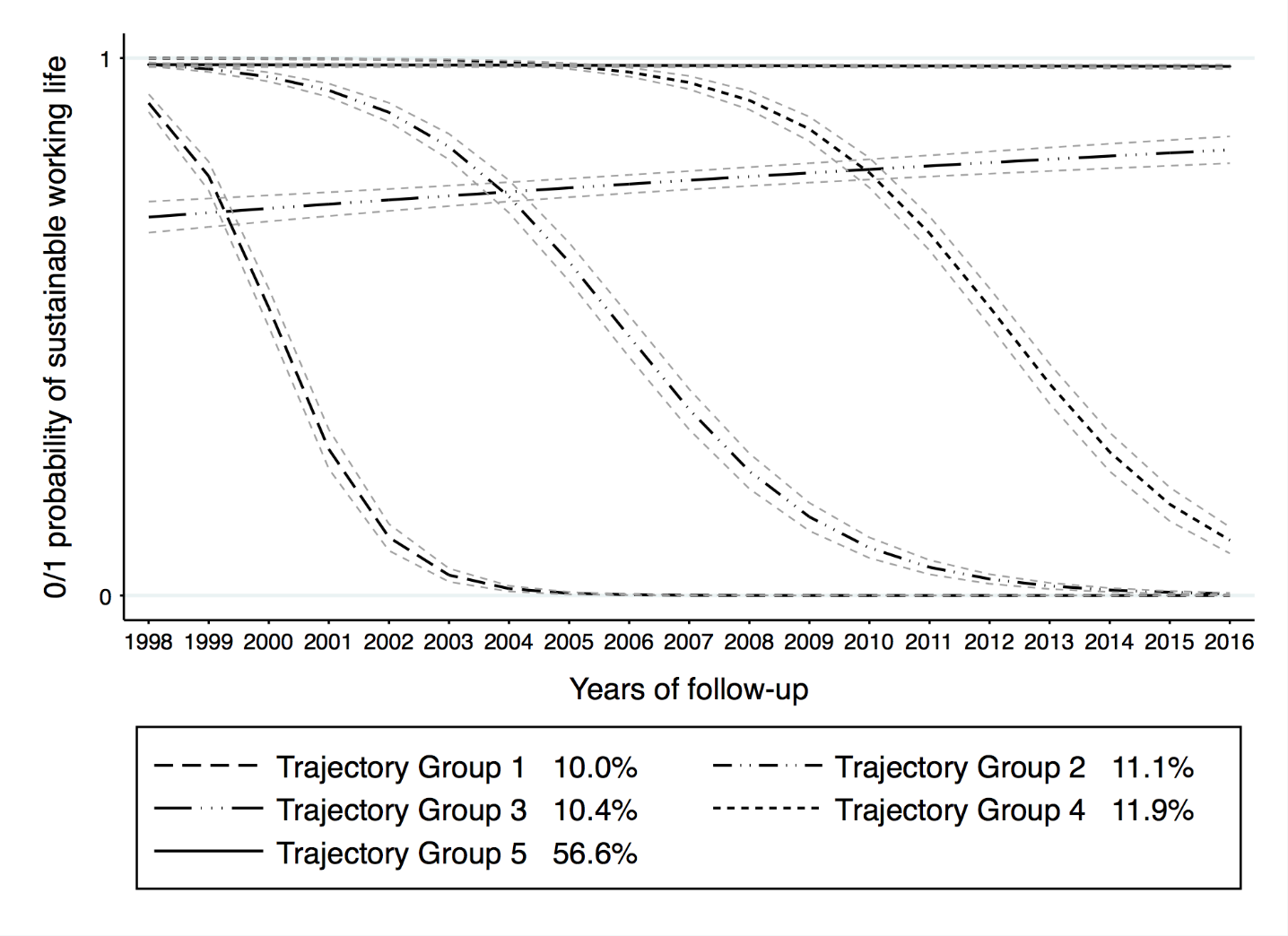


**Supplemental Figure S2** Trajectory groups of sustainable working life in region 5, Commuting municipalities with a low commuting rate near medium-sized towns - municipalities where less than 40% of the working population commute to work in a medium-sized town.


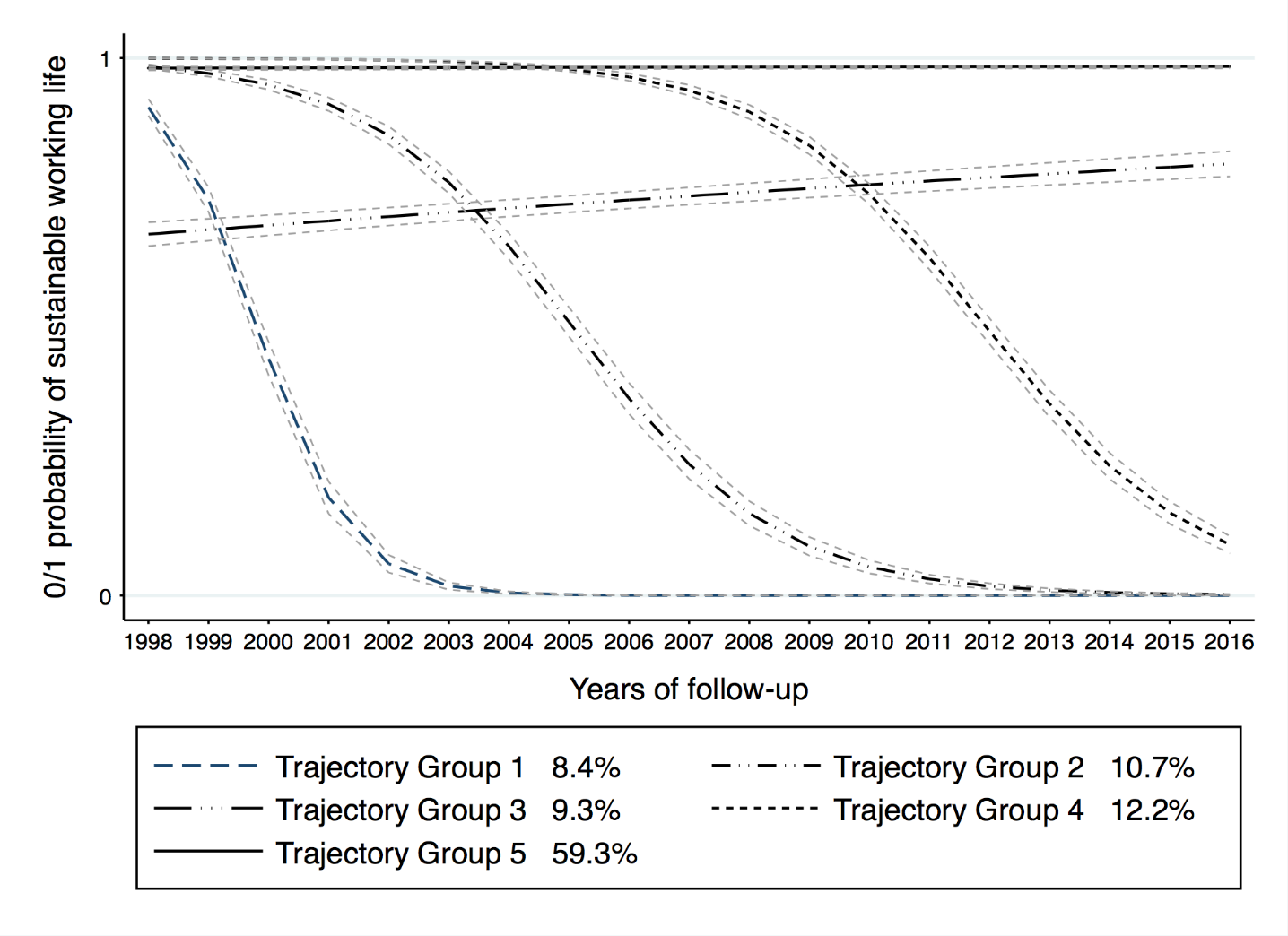


**Supplemental Figure S3** Trajectory groups of sustainable working life in region 6, Small towns - municipalities with a population of at least 15, 000 inhabitants in the largest urban area.


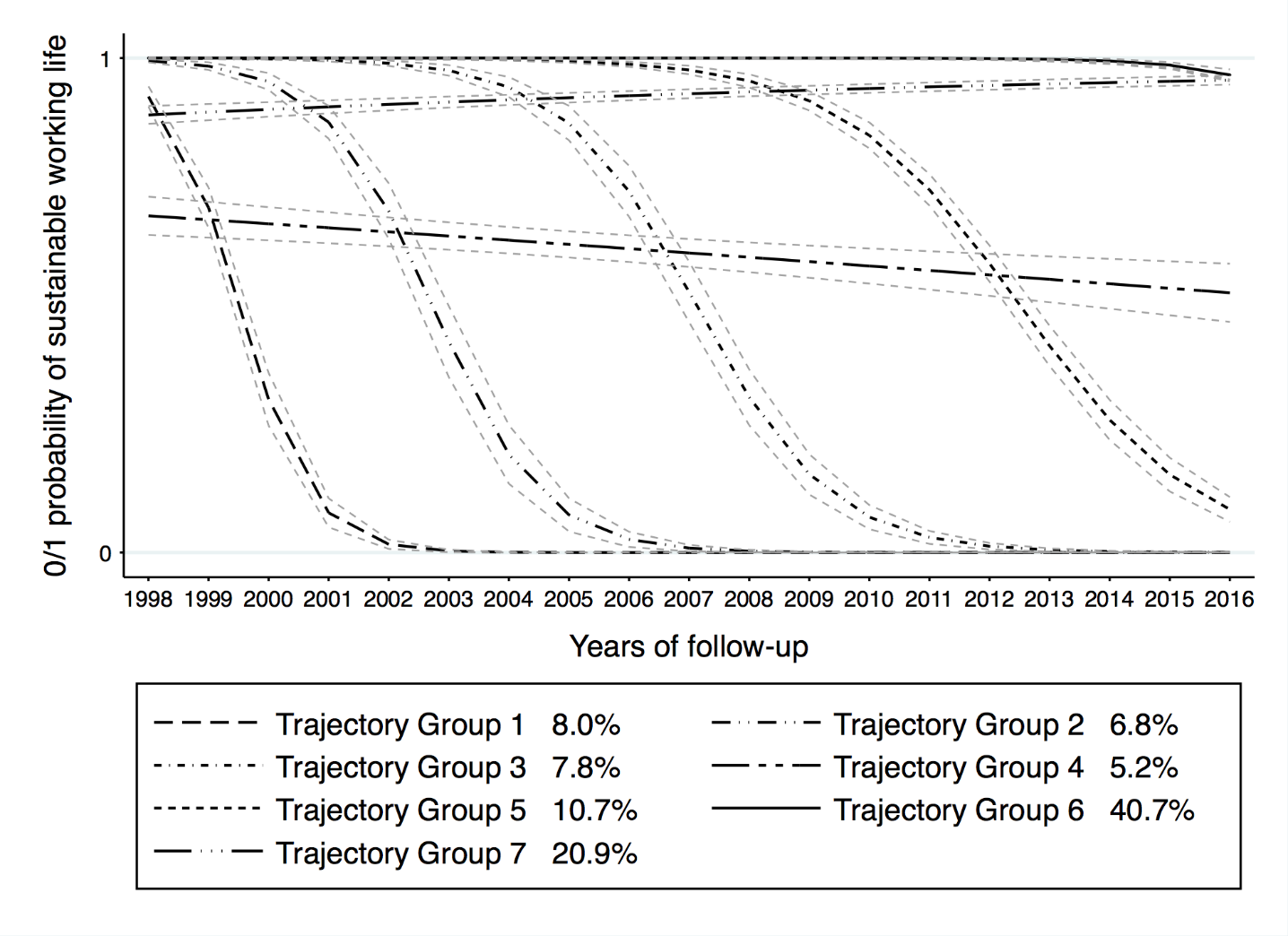


**Supplemental Figure S4** Trajectory groups of sustainable working life in region 7, Commuting municipalities near small towns - municipalities where more than 30% of the working population commute to work in a small town/ urban area or more than 30% of the employed day population lives in another municipality.


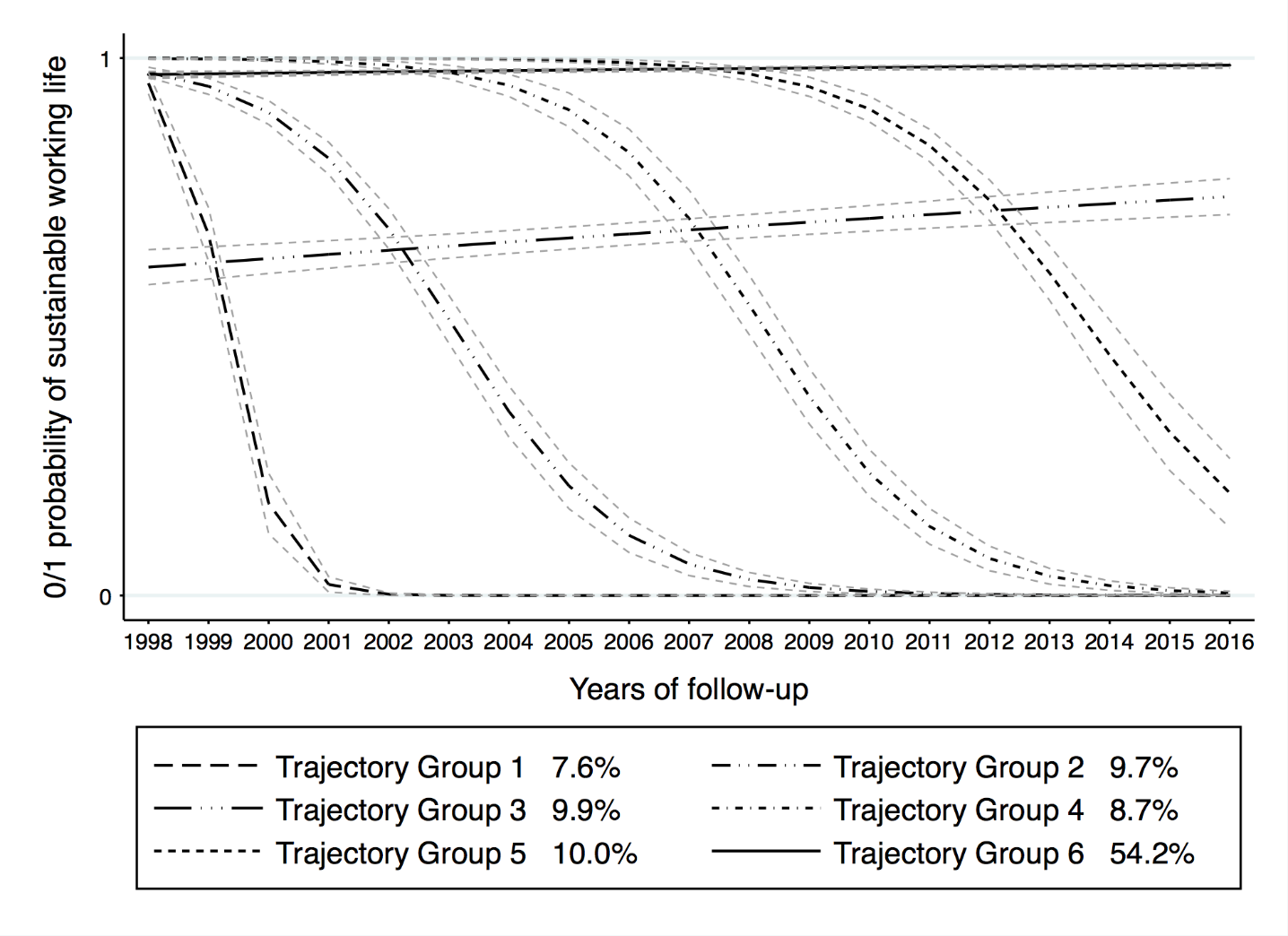


**Supplemental Figure S5** Trajectory groups of sustainable working life in region 8, Rural municipalities - municipalities with a population of less than 15 000 inhabitants in the largest urban area, very low commuting rate (less than 30%).


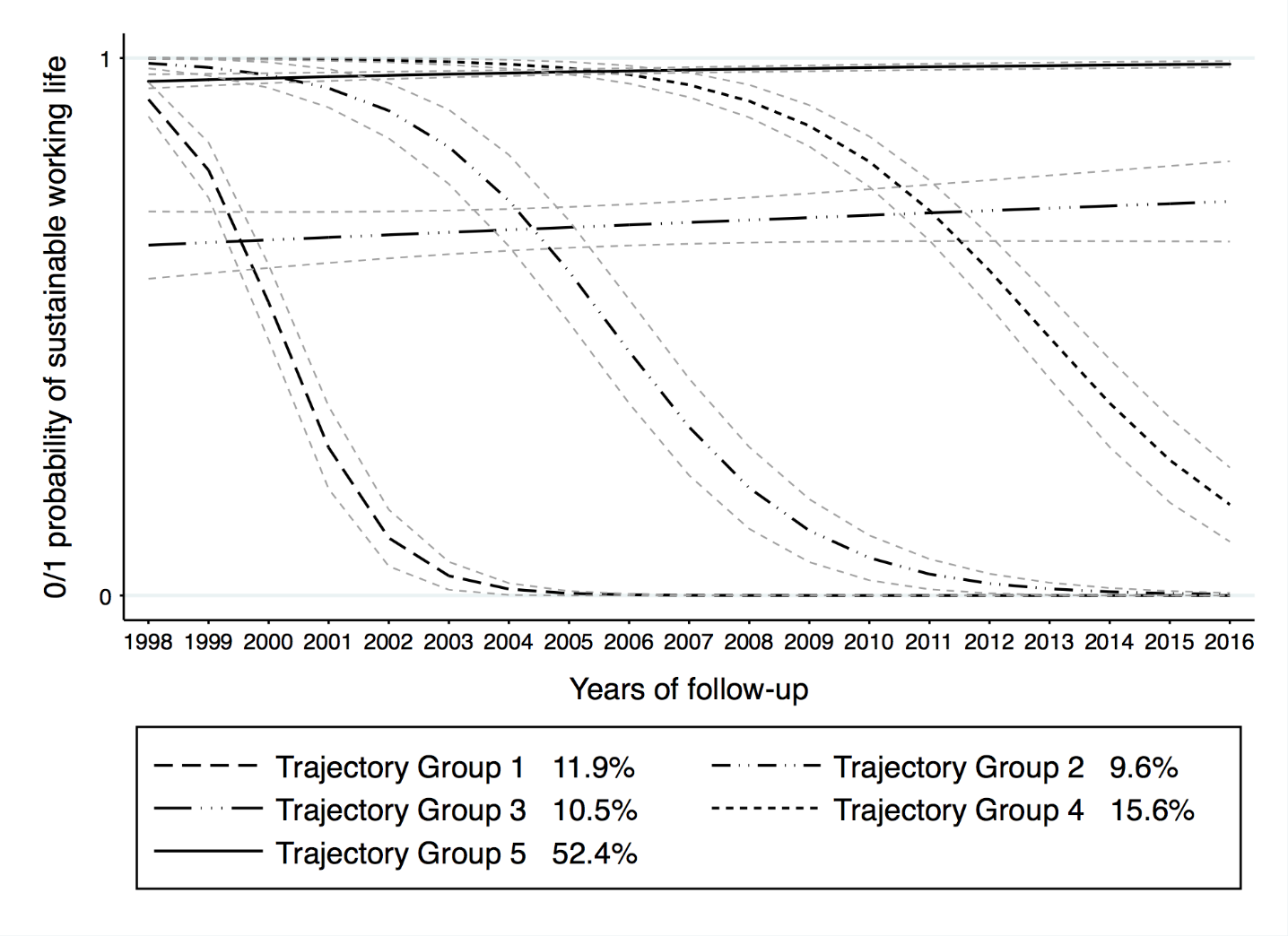


**Supplemental Figure S6** Trajectory groups of sustainable working life in region 9, Rural municipalities with a visitor industry – municipalities in rural area that fulfil at least two criteria for visitor industry, i.e., number of overnight stays, retail-, restaurant- or hotel turnover per head of population.
